# Repeated Restraint Stress Led to Cognitive Dysfunction by NMDA Receptor-Mediated Hippocampal CA3 Dendritic Spine Impairments in Juvenile Sprague-Dawley Rats

**DOI:** 10.3389/fnmol.2020.552787

**Published:** 2020-10-19

**Authors:** Dong-sheng Sun, Gang Zhong, Hong-Xia Cao, Yu Hu, Xiao-Yue Hong, Ting Li, Xiao Li, Qian Liu, Qun Wang, Dan Ke, Gong-ping Liu, Rong-Hong Ma, Dan-Ju Luo

**Affiliations:** ^1^Institute of Anesthesiology & Pain (IAP), Department of Anesthesiology, Taihe Hospital, Hubei University of Medicine, Shiyan, China; ^2^Department of Otorhinolaryngology, Union Hospital, Tongji Medical College, Huazhong University of Science and Technology, Wuhan, China; ^3^Department of Pathology, Tongji Hospital, Tongji Medical College, Huazhong University of Science and Technology, Wuhan, China; ^4^Department of Pathophysiology, School of Basic Medicine and the Collaborative Innovation Center for Brain Science, Key Laboratory of Ministry of Education of China for Neurological Disorders, Tongji Medical College, Huazhong University of Science and Technology, Wuhan, China; ^5^Co-innovation Center of Neuroregeneration, Nantong University, Nantong, China; ^6^Department of Clinical Laboratory, Union Hospital, Tongji Medical College, Huazhong University of Science and Technology, Wuhan, China; ^7^Department of Pathology, Union Hospital, Tongji Medical College, Huazhong University of Science and Technology, Wuhan, China

**Keywords:** chronic stress, cognitive dysfunction, NMDA receptors, CA3 region, hippocampus, synaptic impairment

## Abstract

Although numerous studies have indicated that chronic stress causes cognitive dysfunction with the impairment of synaptic structures and functions, the relationship between cognitive deficits induced by repeated restraint stress and the level of NMDA receptors in the subregion of the hippocampus has been relatively unknown until now. In this study, 3-week-old male Sprague-Dawley rats were exposed to repeated restraint stress for seven consecutive days, their cognitive functions were evaluated through behavioral tests, and then they were sacrificed for electrophysiological, morphological, and biochemical assays. Chronic repeated restraint stress led to cognitive and electrophysiological impairments, with a reduced density of dendritic spines. We also found that the protein level of NMDA receptors only increased in the hippocampal CA3 region. Nevertheless, repeated restraint stress-induced cognitive and synaptic dysfunction were effectively reversed by Ro25-6981, an inhibitor of the GluN2B receptor. These findings suggest that repeated restraint stress-induced synaptic and cognitive deficits are probably mediated through NMDA receptors.

## Introduction

Stress induces dramatic changes in the hippocampal structure and function (McEwen, 2000b; Vyas et al., 2002), as well as severely influences mood and cognitive functions (Kim and Diamond, 2002; de Kloet et al., 2005). Many studies have reported that stress reinforced long-term depression (LTD), a major form of synaptic plasticity-related to learning and memory through *N*-methyl-D-aspartate receptors (NMDA-receptor) in the hippocampal CA1 region (Kim et al., 1996; de Kloet et al., 1999; McEwen et al., 2012). A complex series of prominent cellular and molecular changes occur in the brain exposed to chronic stress, which is associated with cognitive dysfunction (Nasca et al., 2015; Zhou et al., 2018). Chronic stress leads to cognitive deficits and growth inhibition of neuronal dendrites, while the deleterious effects caused by chronic stress are reversed after phosphatase PDE2 inhibition (Xu et al., 2015). Some studies have reported that chronic repeated stress suppressed the neurogenesis in the hippocampal dentate gyrus (DG), reduced hippocampal DG long-term potentiation (LTP), and induced atrophy of hippocampal DG, as well as resulted in shrinkage and debranching of dendrites in the hippocampal CA3 region (McEwen, 2000a; Nasca et al., 2015; Zhou et al., 2018). Further studies showed that chronic restraint stress induced the retraction of the apical dendrite involved in the modulation of NMDA receptors in the hippocampal CA3 region (Christian et al., 2011), increased hippocampal glutamate uptake and release (Fontella et al., 2004), and enhanced metabotropic glutamate receptor-mediated LTD (Sengupta et al., 2016). In addition, chronic restraint stress was reported to induce atrophy of the CA1 hippocampus (Sandi and Pinelo-Nava, 2007; Lupien et al., 2009), reduce hippocampal CA1-LTP (Bhagya et al., 2017), and cause apoptosis of neurons and the reduction of dendritic spine density in the hippocampus CA1 region (Huang et al., 2015).

The *N*-methyl-D-aspartic acid receptor is a subtype of the ionotrophic glutamate receptor and is principally distributed in the neuronal postsynaptic membrane (McBain and Mayer, 1994). It is made up of two subunits: NMDA-receptor subunit NR1 (GluN1) and NMDA-receptor subtype 2 (GluN2) that contain multiple subtypes (GluN2A, GluN2B, GluN2C, and GluN2D) (Randall and Thayer, 1992; McEwen, 2000b). The NMDA receptor not only plays a key role in the function of the nervous system involved in the regulation of neuron growth and excitability as well as mediation of neurite growth and synaptic plasticity, but is also closely associated with the formation of neural circuits (Olney, 1986; McEwen, 2000a; Xu et al., 2015), which is of vital importance in the processes of learning and memory (Tsien, 2000). The NMDA receptor is also crucial for dynamic maintenance of synaptic stability correlating with the preservation of remote memories (Cui et al., 2004). However, a high glutamate level causes over-activation and overexpression of the NMDA receptor; and impairs the structure and function of the brain (Olney, 1986). Many studies have found that over-activation of the NMDA receptor causes excitatory toxicity, which results in neuronal death and degeneration (Donohue et al., 2006; Huang et al., 2015; Bhagya et al., 2017). Consequently, NMDA receptor-mediated excitatory neurotoxicity may play a vital role in cognitive impairment and neuronal synaptic degeneration correlating with stress. However, the relationship between cognitive deficits induced by repeated restraint stress and the level of NMDA receptors in the subregion of the hippocampus has not been clear until now.

In the present study we investigated the effect of chronic repeated restraint stress on Sprague-Dawley rats (SD, 3-week-old) for seven consecutive days on the structure and function of the hippocampal regions and its underlying molecular mechanism. We found that repeated restraint stress exposure led to cognitive dysfunction with reduced dendritic spines density and LTP, and enhancement of LTD. NMDA receptor protein levels increased only in the CA3 region of the hippocampus. Nevertheless, these cognitive and LTD deficits were partially reversed by treatment with a GluN2B inhibitor.

## Materials and Methods

### Animals

We obtained 3-week-old male SD rats (weighing 100 ± 20 g) from the Laboratory Animal Center of Huazhong University of Science and Technology. All rats were housed in polypropylene cages and kept in a stable ambient temperature (23 ± 1°C) with a regulated 12/12 h (light/dark) cycle. Food and drink were available *ad libitum*. The animal experimental procedure was conducted according to the “Policies on the Use of Animals and Humans in Neuroscience Research,” and approved by the Ethics Committee of Tongji Medical College, Huazhong University of Science and Technology.

### Repeated-Stress Procedure

The repeated-stress procedure on rats was conducted as previously described (Mozhui et al., 2010). Briefly, rats were placed in air-assessable cylinders (the container size was similar to the animal size, which made the animal almost immobile in the container) for 2 h daily (14:00 to 16:00) for seven consecutive days. The control rats were not exposed to chronic repeated stress and remained undisturbed in the home cage.

### Morris Water Maze (MWM) Test

The learning and spatial memory abilities of rats were assessed by the Morris water maze (MWM) test 24 h after stress exposure cessation. A circular pool (60 cm high and 160 cm in diameter) full of water (22 cm in depth) was used for the MWM test. The water in the pool was opacified by using dry milk and the water temperature was kept at 22–24°C. The pool was virtually divided into four quadrants. A circular platform (10 cm diameter) was submerged 1.5 cm below the water level in a fixed point in one of the quadrants (Platform quadrant). For spatial learning, rats were trained to search the platform hidden in water for 5 d (four trials per day) in a row from 14:00 to 20:00. Experimental operators let the rats face the wall of the pool and start from one of the four quadrants, and allowed the animals to search for the platform for 60 s in each trial. If the animals climbed onto the hidden platform in the allotted time, the training trial was automatically terminated, and then the rats stayed there for 30 s. If the animals could not find the hidden platform at the allocated time (60 s), the operators guided them to the platform and kept them there for 30 s. The escape latency and swimming path of each rat were recorded using a video camera (Ethovision, Noldus Information Technology, Beijing, China) fixed to the ceiling. For spatial memory, the operators removed the platform and the time spent in each quadrant and the total times crossing the place where the escape platform was located were recorded.

### Novel Object Recognition (NOR) Test

The recognition memory of the rats was assessed using the novel object recognition (NOR) test. Briefly, the rats were placed in the arena to explore two identical objects (cylindrical) located in opposite corners of the arena for 10 min in the acquisition trial. After 24 h, the novel object test was performed. One of the two cylindrical objects was replaced with a cubical object (novel object). The animals were permitted to explore both objects for 10 min. An overhanging camera connected to a computer was used to record the behavior and the exploration time on each object for each rat, and object investigation ratio (the time spent exploring the novel object or familiar object divided by the total 10 min during the test trial), discrimination ratio (the difference in time spent exploring the novel and familiar objects divided by the total time spent exploring both objects during the test trial), and exploration time (the total exploration time on both objects during acquisition trial) were analyzed using a software system.

### Fear Condition (Fear Memory) Test

A fear condition test was performed as described previously in our lab (Jin et al., 2017). The animals were placed in a sound-attenuated chamber with a metal grid floor. In the training phase (1st day) each animal was placed in the chamber for 3 min to habituate the testing environment and then three consecutive foot shocks with 1-min interval (0.5 mA, 2 s duration) was delivered. After the last foot shock was completed, the rat was returned to the cage. In the fear conditioning testing phase (2nd day), each rat was placed in the same chamber and stayed there for 3 min without any stimulus. A video camera fixed onto the chamber was used to record the freezing time (freezing, defined as a lack of movement except for heart beat and respiration, associated with a crouching posture). The freezing time was measured by software. The ratio of freezing time during the training/test phase was defined as the freezing time divided by the total time during the training/test phase.

### Electrophysiological Measure

Rats were deeply anesthetized and the brain was removed, following which coronal slices (300-μm thick) were cut by a Leica vibratome in ice-cold artificial cerebrospinal fluid (aCSF) containing: NaCl 126 mM, KCl 3.0 mM, MgCl_2_ 1.0 mM, CaCl_2_ 2.0 mM, NaH_2_PO_4_ 1.25 mM, NaHCO_3_ 26 mM, and glucose 10 mM, saturated with 95% O_2_, 5% CO_2_ (pH 7.4). Afterward, the slices were transferred into an incubation chamber filled with oxygenated aCSF to recover for 1 h before measurement. Slices were laid down in a chamber with an 8 × 8 microelectrode array in the bottom planar (each 50 × 50 μm in size, with an interpolar distance of 450 μm) and kept submerged in aCSF (4 mL/min) with nylon silk glued to a platinum ring. The MED64 system (Alpha MED Sciences, Panasonic, Osaka, Japan) was used to acquire signals as described in the previous report (Li et al., 2019). The field excitatory postsynaptic potentials (fEPSPs) were elicited by stimulating the mossy fiber in the dentate hilus and recorded in CA3. Stimulation intensity was adjusted to evoke fEPSP slopes that were 40% of the maximal size. LTP was induced by applying one train of high-frequency stimulation (HFS; 100 Hz, 1 s duration at test strength). LTD was induced by applying a 900 train of low-frequency stimulation (1 Hz, 15 min duration at test strength). AP-5 (2-amino-5-phosphopentanoic acid, an NMDA receptor inhibitor, Sigma) 50 μM, PEAQX ([[[(1S)-1-(4-Bromophenyl) ethyl] amino] (1,2,3,4-tetrahydro-2,3-dioxo-5-quinoxalinyl)methyl] phosphonic acid tetrasodium hydrate, a GluN2A inhibitor, Sigma) 0.5 μM, or Ro25-6981 (GluN2B inhibitor, Sigma) 0.5 μM was present in aCSF 10 min before and during LFS stimulation.

### Microinfusion

To investigate the effect of Ro25-6981 on cognition, rats were microinfused with Ro25-6981 in the CA3 region 30 min before restraint stress. For the microinfusion experiments, guide cannulas were implanted into the CA3 region bilaterally. After implantation, the guide cannula was secured to the skull with three skull screws and dental acrylic and closed with an obturator. Ro25-6981 (0.5 μM) or equal volume of normal saline was administered by stereotactic intracerebral injection (1 μl for 5 min) via a guide cannula and retained for 10 min after injection. Following restraint stress, the rats were subjected to the novel object recognition and fear condition test.

### Golgi Staining

The effect of exposure to repeated restraint stress on the morphology of hippocampal neurons were investigated by Golgi-Cox impregnation using a GD Rapid Golgi Stain Kit (FD NeuroTechnologies). Briefly, the rats were deeply anesthetized and perfused with 500 ml of phosphate buffer saline. After 5 min, brains were quickly removed, and then immersed in impregnation solution (equal volumes of solutions A and B, containing HgCl_2_, Cr_2_K_2_O_7_, and CrK_2_O_4_) and stored at 20°C. The impregnation solution was replaced every 2 days. After 3 weeks, brains were dehydrated in 30% sucrose solution for 5 d. A Leica vibrates microtome was used to section the brain sagittally (50 μm), and the sections were mounted on gelatin-coated microscope slides. The slides were immersed in distilled water for 1 min, ammonia solution for 30 min in the dark, Kodak fixing solution for 30 min in the dark, distilled water for 1 min, 50%, 70%, and 95% alcohol for 1 min each, 100% alcohol for 5 min (three times), and CXA solution (xylene:chloroform:anhydrous ethanol = 1:1:1) for 5 min in turn. Subsequently, the sections were sealed with neutral balsam and dried in a fume hood. A light microscope (Nikon) was used to image the sections.

The density of the spine was measured on apical tertiary dendritic segments of the CA1 and CA3 pyramidal neuron, and tertiary dendrites of DG neurons. For the spine analysis, a Zeiss AxioImager microscope with a 63× oil immersion objective was used to acquire the images of the dendritic segments, and a previously reported method in our lab was used to analyze the spine morphology (Jin et al., 2018). Though it could not assess the spine density in a 3-dimensional manner, the method focused on spines parallel to the plane of sectioning. When treatment groups were analyzed identically, the method facilitated a direct comparison, though the total number of spines may have been underestimated. The linear spine density, presented as the number of spines per 10 μm of dendrite length, was calculated by the Image J software. Data from 5 to 7 neurons per rat were averaged and used for statistical analysis.

### RNA Extraction and Quantitative Real-Time PCR

TRIzol reagents were used to isolate total RNA from the fresh hippocampus of rats according to the manufacturer’s instructions (Invitrogen, Carlsbad, CA, United States) and a reverse transcription reagent kit (Takara, Dalian, China) was used to transcribe RNA into cDNA. The produced cDNA (2 μl) was used for real-time PCR with primer: 5′-AGAGCCCGACCCTAAAAAGAA-3′ and 5′-CCCTCCTCCCTCTCAATAGC-3′ for GluN1, 5′-TGAGAATTGCTCGGTGTCTG-3′ and 5′-ACCTGGCACTGTAG GAATGG-3′ for GluN2A, 5′-GGCTACGGCTACACATGGAT-3′ and 5′-CCTCT TCTCGTGGGTGTTGT-3′ for GluN2B, and 5′-TGTTACCAACTGGGACGACA-3′ and 5-ACCTGGGTCATCTTTTCACG-3′ for β-actin.

### Western Blotting

The rats were decapitated under deep anesthesia and the tissue of hippocampal CA1, CA3, or DG regions were quickly dissected from the brain on ice. The hippocampal tissues were homogenized in ice-cold buffer (containing Tris 10 mM, NaCl 50 mM, Na_3_VO_4_ 0.5 mM, NaF 50 mM, EDTA 1 mM, and PMSF 1 mM) plus a protease-inhibitor cocktail (Sigma P8340). Later, the extract was sonicated 15 times by a probe-type sonicator after boiling for 10 min. The BCA protein assay kit (Pierce) was used to measure the protein concentration of the sample. The samples were stored at −80°C.

For western blotting, an equal quantity of protein from each sample was subjected to 10% SDS-PAGE. Subsequently, the proteins were transferred onto nitrocellulose membranes and blocked with non-fat milk (5%) for 1 h. The blots were incubated with primary antibodies listed in Table 1 at 4°C for 12 h, and then incubated with goat anti-mouse or anti-rabbit IgG conjugated to IRDye (diluted as 1:10,000, 800CW, Licor Biosciences, United States) for 1 h at room temperature. Finally, an Odyssey infrared imaging system (Li-Cor Biosciences, United States) was used to visualize and analyze the immunoreactive bands.

**TABLE 1:** Antibodies used in the study.

**Antibody**	**Specific**	**Type**	**WB**	**Source**
GluN2A	*N*-methyl-D-aspartate receptor subtype 2A	pAb	1:1,000	Abcam
GluN2B	*N*-methyl-D-aspartate receptor subtype 2B	pAb	1:1,000	Abcam
GluN	*N*-methyl-D-aspartate receptor subunit NR1	mAb	1:1,000	Millipore
GluA1	AMPA-selective glutamate receptor 1	pAb	1:1,000	Thermo
GluA2	AMPA-selective glutamate receptor 1	mAb	1:1,000	Abcam
Pan	*C*-terminus of human Cadherins	mAb	1:500	Abcam
DM1A	α-tubulin	mAb	1:1,000	Sigma

### Statistical Analysis

The data were expressed as mean ± SEM or mean ± SD and analyzed by one-way analysis of variance followed by Bonferroni’s *post hoc* tests or Student’s unpaired *t*-test with the SPSS version 18.0 statistical software (SPSS Inc., Chicago, IL, United States). Statistical significance was considered as a *p*-value of < 0.05.

## Results

### Repeated Restraint Stress Led to Cognitive Dysfunction in SD Rats

**FIGURE 1:**
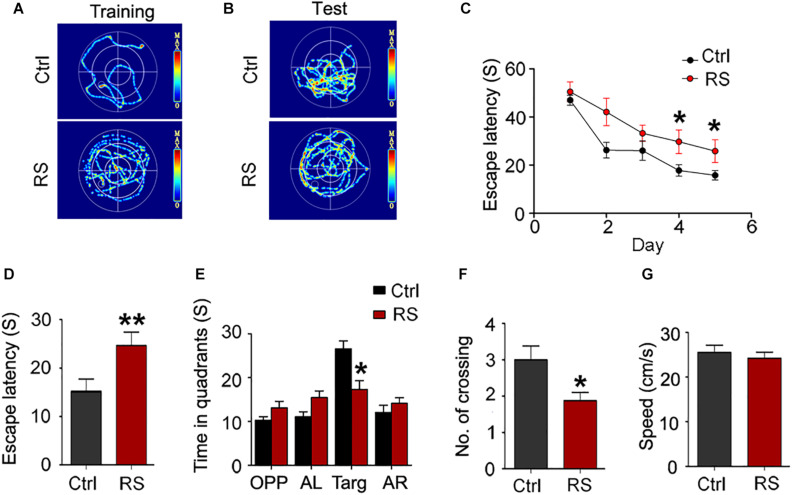
Repeated restraint stress exposure led to cognitive deficits detected by the MWM test. The representative swimming trace of the rats during training **(A)** and memory test **(B)** in the water maze. Escape latency (time taken to escape onto the platform) during training **(C)**. During test, escape latency **(D)**, the time spent in quadrant **(E)**, and the numbers crossing the site where the platform placed (No. of crossing) **(F)**. The swimming speed (mean swimming speed of days 1–5) of the control and rats exposed to repeated stress in the MWM test **(G)**. OPP, opposite quadrant; AL, adjacent left quadrant; Targ, target quadrant; AR, adjacent right quadrant. All data were expressed as mean ± SEM (*n* = 8 rats each group). *p < 0.05, **p < 0.01 vs Ctrl.

To investigate the effects of repeated restraint stress on learning and memory functions, the MWM test was first performed 24 h after stress exposure cessation. The representative images of swim trace during the training or test phase are shown as Figures 1A and B. We found that escape latency (time taken to escape onto the platform) in rats exposed to restraint stress significantly increased on the fourth and fifth day compared with the control during the training trial (Figure 1C), indicating that repeated restraint stress exposure weakened the learning ability of the animals. Similarly, repeated restraint stress notably increased the escape latency to find the target zone when the platform was located earlier (Figure 1D), as well as significantly decreased the number of crossing and the time spent in the target quadrant during the memory probe test phase (Figures 1E,F). The swimming speed (mean swimming speed of days 1–5) had no significant change (Figure 1G), which meant that the animals had no motor deficits.

**FIGURE 2:**
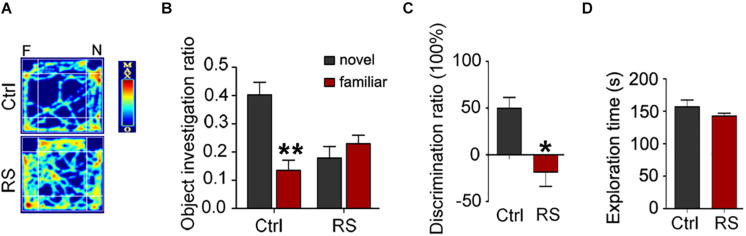
Repeated restraint stress exposure impaired recognition memory detected by the NOR test. The representative heat map for the rats moving in novel object recognition test **(A)**. F, the familiar object; N, the novel object. The object investigation ratio (the time spent in exploring the novel object or familiar object divided by the total 10 min during the test trial) **(B)** (**p < 0.01 vs Novel), discrimination ratio (the difference in time spent in exploring the novel and familiar objects divided by the total time spent in exploring both objects during the test trial) **(C)**, and exploration time (the total exploration time on both objects during acquisition trial) **(D)**. All data were expressed as mean ± SEM (*n* = 8 rats each group). *p < 0.05 vs Ctrl.

**FIGURE 3:**
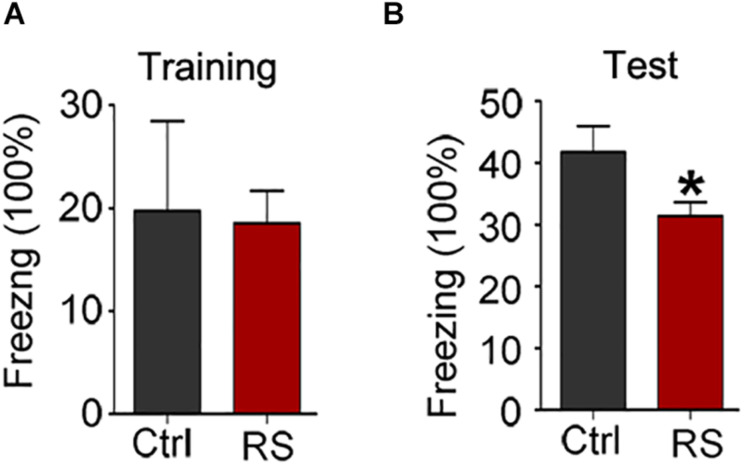
Repeated restraint stress impaired fear memory ability detected by contextual fear condition test. The ratio of freezing time during training phase **(A)** and test phase **(B)**. All data were expressed as mean ± SEM (*n* = 8 rats each group). *p < 0.05 vs Ctrl.

To further prove repeated restraint stress-induced cognitive deficits, we next conducted a novel object recognition and fear conditioning test. We found that repeated restraint stress exposure impaired the recognition memory as revealed by a NOR test, which showed a significant decrease in the time exploring the novel object (the object investigation ratio and discrimination ratio) and no difference in the exploration time in repeatedly stressed rat compared to control rats (Figure 2). Meanwhile, the freezing time was significantly reduced in the animals administrated with repeated restraint stress (Figure 3), which was measured by the contextual fear conditioning test. These findings suggest that repeated restraint stress induces learning and memory deficits.

### Repeated Restraint Stress Increased the Expression of NMDA Receptors in the Hippocampal CA3, but Not CA1 and DG Regions

**FIGURE 4:**
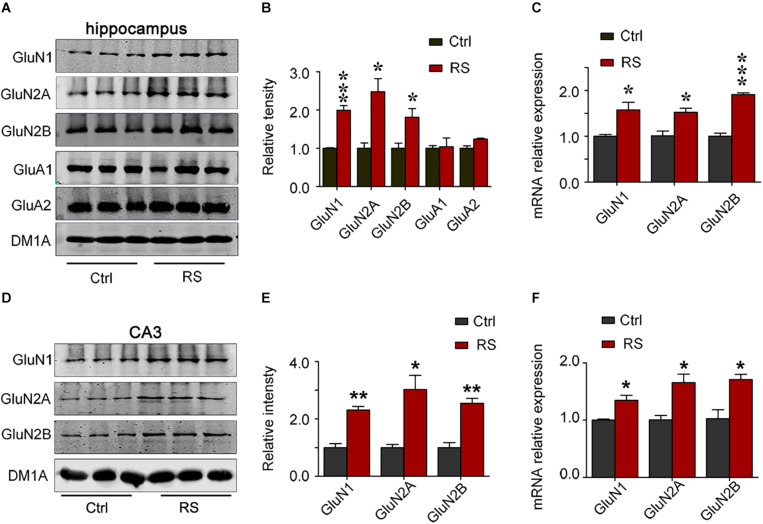
Repeated restraint stress increased NMDA receptor protein levels in the hippocampal CA3 region. The protein and mRNA levels of *N*-methyl-D-aspartate receptor subtype 1 (GluN1), *N*-methyl-D-aspartate receptor subtype 2A (GluN2A), *N*-methyl-D-aspartate receptor subtype 2B (GluN2B), AMPA-selective glutamate receptor 1 (GluA1), and AMPA-selective glutamate receptor 2 (GluA2) in the hippocampus estimated by western blotting **(A,B)** or qRT-PCR **(C)** methods, respectively. The total protein or mRNA levels of *N*-methyl-D-aspartate receptor subtype 1 (GluN1), *N*-methyl-D-aspartate receptor subtype 2A (GluN2A), and *N*-methyl-D-aspartate receptor subtype 2B (GluN2B) in the hippocampal CA3 region estimated by western blotting **(D,E)** or qRT-RCR **(F)** methods, respectively. Relative intensity is expressed as the level of each protein against the level of DM1A, which was quantified using the Image J software. Data were expressed as mean ± SD (*n* = 3 rats each group). *p < 0.05, **p < 0.01, ***p < 0.001 vs Ctrl.

To investigate the molecular mechanisms underlying the repeated restraint stress-induced cognitive dysfunction, we measured NMDA receptor expression in the hippocampus of rats. The total level of GluN1, GluN2A, or GluN2B was significantly increased in the hippocampus of rats exposed to repeated restraint stress, compared with the control (Figures 4A,B). Similarly, mRNA levels of the NMDA receptors were also increased (Figure 4C) under repeated restraint stress conditions. Further we measured NMDA receptor expression in different hippocampal regions. We found that repeated restraint stress significantly increased the total protein and mRNA levels of GluN1, GluN2A, and GluN2B in the hippocampal CA3 region (Figures 4D–F).

**FIGURE 5:**
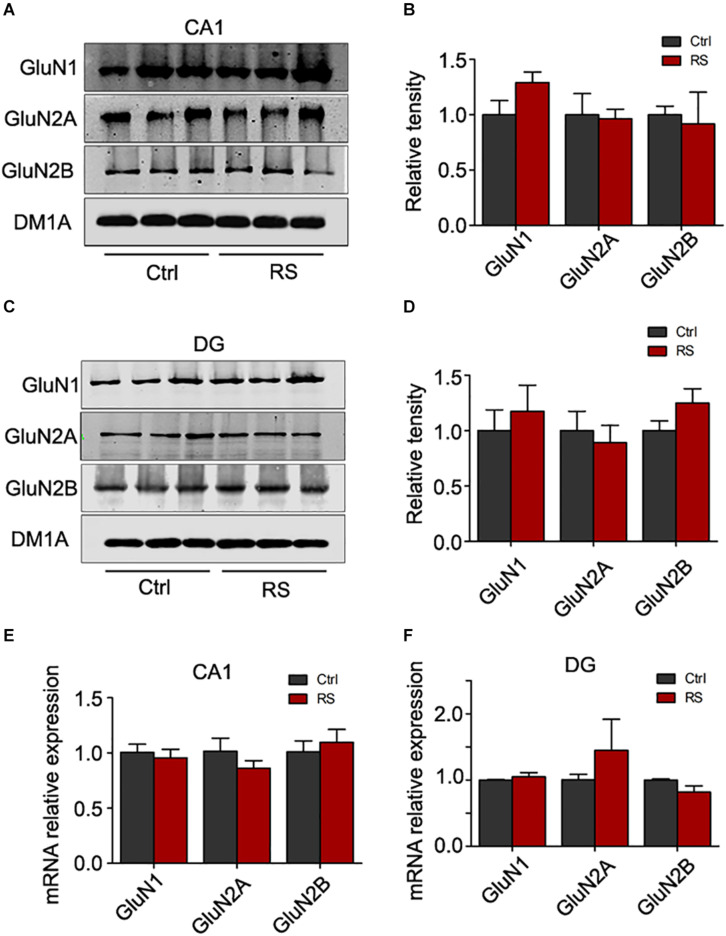
Repeated restraint stress failed to change the protein level of NMDA receptors in the hippocampal CA1 and DG regions. The protein levels of *N*-methyl-D-aspartate receptor subtype 1 (GluN1), *N*-methyl-D-aspartate receptor subtype 2A (GluN2A), and *N*-methyl-D-aspartate receptor subtype 2B (GluN2B) in the hippocampal CA1 **(A,B)** or DG **(C,D)** region estimated by western blotting. The total mRNA levels of *N*-methyl-D-aspartate receptor subtype 1 (GluN1), *N*-methyl-D-aspartate receptor subtype 2A (GluN2A), and *N*-methyl-D-aspartate receptor subtype 2B (GluN2B) in the hippocampal CA1 **(E)** or DG **(F)** region estimated by qRT-PCR methods. Relative intensity is expressed as the level of each protein against the level of DM1A, which was quantified using the Image J software.

In contrast to the hippocampal CA3 region, stress did not significantly alter the protein (Figures 5A–D) and mRNA levels (Figures 5E,F) of NMDA receptors in the hippocampal CA1 and DG regions. These data suggest that repeated restraint stress specifically influenced the level of NMDA receptors in the CA3 region.

### Dendritic Plasticity Was Impaired in the Hippocampal CA3 Region by Repeated Restraint Stress

**FIGURE 6:**
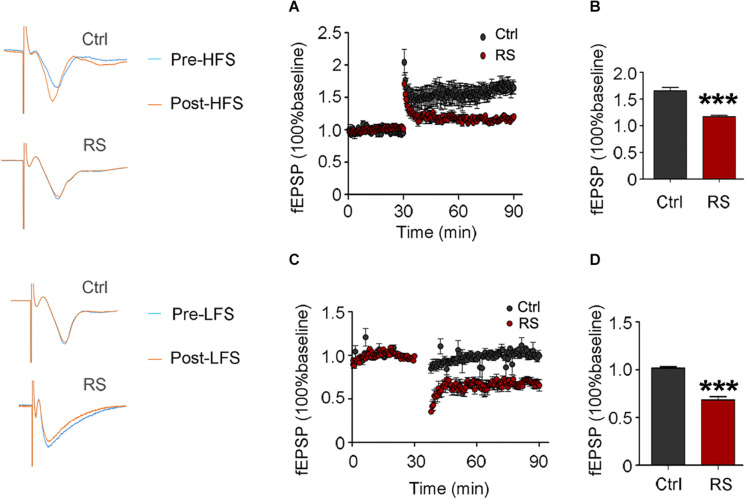
Repeated restraint stress attenuated LTP and enhanced LTD. Effects of repeated restraint stress exposure in LTP, which was elicited by stimulating the mossy fiber in the dentate hilus and recorded in CA3 **(A)**, and quantitative analyses for normalized fEPSPs 80–90 min after HFS **(B)**. Effects of repeated stress exposure in LTD **(C)**, and quantitative analyses for normalized fEPSPs 80–90 min after LFS **(D)**. Data were expressed as mean ± SD (*n* = 4 rats each group). ***p < 0.001 vs Ctrl.

The increased protein level of the NMDA receptor may induce excitatory neurotoxicity. In order to explore the neurobiological mechanism of the repeated restraint stress-induced cognitive dysfunction, we measured LTP and LTD in CA3 by stimulating the mossy fiber of dentate hilus in acute hippocampal slices. Compared with the control, a significantly attenuated LTP was detected in the rats exposed to repeated restraint stress after HFS (Figures 6A,B). Additionally, repeated restraint stress induced a significantly enhanced LTD after low-frequency stimulation (Figures 6C,D).

**FIGURE 7:**
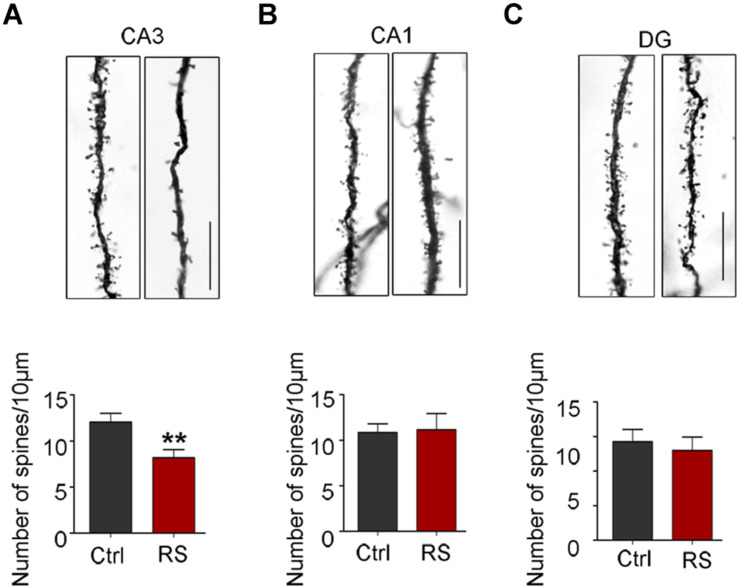
Repeated restraint stress decreased the density of dendritic spines in the hippocampal CA3 region. Representative dendritic spine images and quantitative analyses of dendritic spine density in the hippocampal neurons of CA3 **(A)**, CA1 **(B)**, or DG regions **(C)**. Scale bars = 10 μm. Data were expressed as mean ± SD (5 to 7 neurons per rat, four rats each group). **p < 0.01 vs Ctrl.

The spines of the dendritic branches are the structural and functional units, which are tightly associated with learning and memory function. Thus, Golgi impregnation was used to verify the structural changes after exposure to restraint stress. We found that the dendritic spine density in the hippocampal CA3 region significantly decreased in rats exposed to repeated restraint stress compared with the control (Figure 7A), while they had no significant change in the CA1 and DG areas (Figures 7B,C). These findings suggest that repeated restraint stress impaired dendrite spine plasticity.

### Repeated Restraint Stress-Induced LTD Enhancement in the Hippocampal CA3 Region and Cognitive Dysfunction Was Blocked by an Inhibitor of GluN2B

**FIGURE 8:**
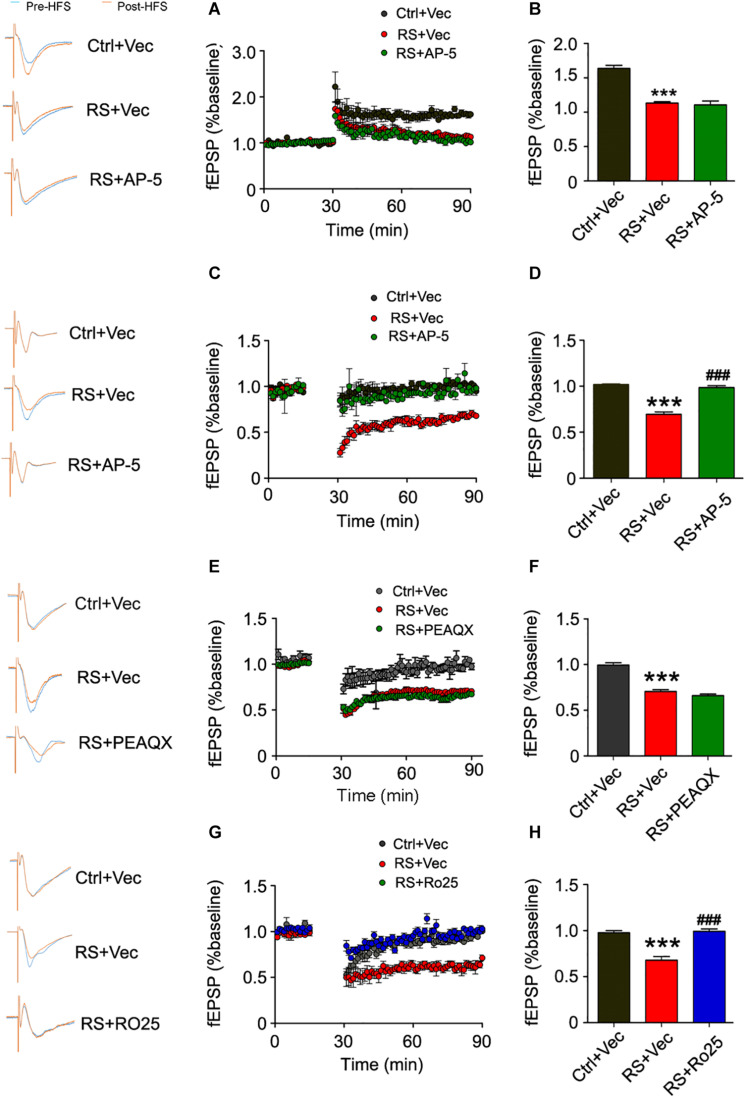
Repeated restraint stress-induced enhancement of LTD was blocked by AP-5 or Ro25-6981. Pretreating with NMDAR antagonist AP-5 **(A)** failed to rescue the attenuation of LTP in slices from rats exposed to repeated stress, and quantitative analyses **(B)** for normalized fEPSPs 80–90 min after HFS. The facilitation of LTD was blocked in slices from rats exposed to repeated stress by pretreating with AP-5 **(C,D)** or selective GluN2B antagonist Ro25-6981 **(G,H)**. Pretreating with GluN2A antagonist PEAQX had no effect on LTD in slices from rats exposed to repeated stress **(E,F)**. All data were expressed as mean ± SD (*n* = 4 rats each group). Ctrl + Vec: pretreating slice from control rats with vehicle; RS + Vec: pretreating slice from rats exposed to repeated stress with vehicle; RS + AP-5: pretreating slice from rats exposed to repeated stress with AP-5; RS + PEAQX: pretreating slice from rats exposed to repeated stress with PEAQX; RS + Ro25: pretreating slice from rats exposed to repeated stress with Ro25-6981. ***p < 0.001 vs Ctrl; ^###^*p* < 0.001 vs RS.

To ascertain which subunit of the NMDA receptor was responsible for repeated restraint stress-induced synaptic impairments, we again measured LTP and LTD at the mossy fiber-CA3 circuit of a pre-intervention brain slice from rats exposed to chronic restraint stress with AP-5 (NMDA receptor inhibitor), PEAQX (GluN2A inhibitor), or Ro25-6981 (GluN2B inhibitor). We found that AP-5 (NMDA receptor inhibitor) pre-intervention failed to ameliorate the impairments of LTP induced by HFS (Figures 8A,B), while repeated restraint stress-induced enhancement of LTD was blocked after AP-5 pre-intervention (Figures 8C,D). In addition, we further found that the inhibitor of GluN2B (Ro25-6981), but not GluN2A (PEAQX) pre-intervention, blocked the enhancement of repeated restraint stress-induced LTD (Figures 8E–H).

**FIGURE 9:**
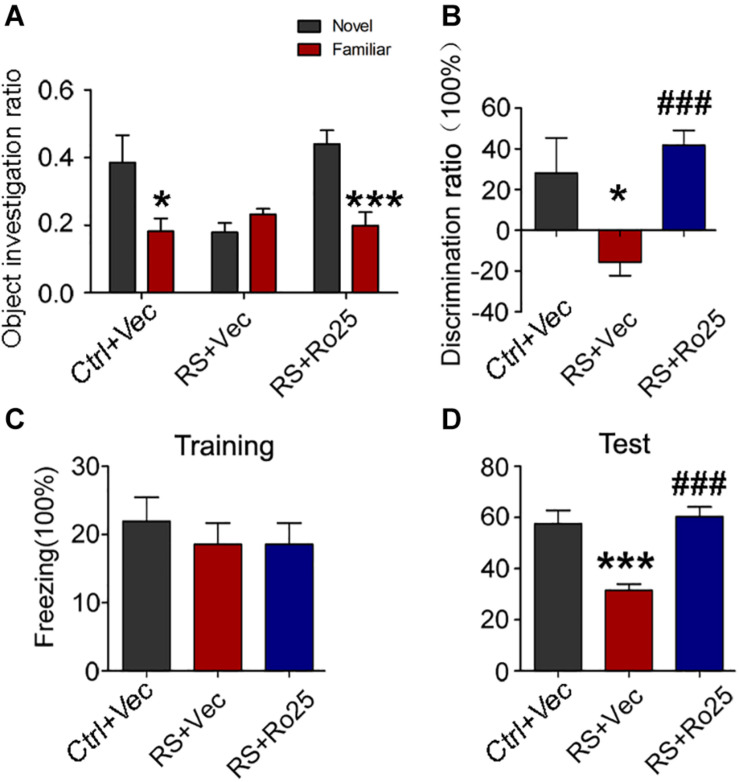
Repeated restraint stress-induced memory deficits were reversed by the inhibition of GluN2B. The object investigation ratio **(A)** and discrimination ratio **(B)** of rats during a novel object recognition test. The freezing time ratio of rats during the training phase **(C)** and test phase **(D)** detected by a contextual fear condition test. All data were expressed as mean ± SEM (*n* = 8 rat each group). Ctrl: rats untreated + vehicle; RS + Vec: rats injected with vehicle and exposed to repeated stress; RS + Ro25: rats injected with Ro25-6981 and exposed to repeated stress. *p < 0.05, ***p < 0.001 vs Ctrl + Vec; ^###^*p* < 0.001 vs RS + Vec.

We further detected the effects of Ro25-6981 (GluN2A inhibitor) on cognitive defects. Results showed a significant increase in the ratio of time spent on novel object exploration and freezing time of the repeated restraint stressed rats, compared with control rats. Thus Ro25-6981 can ameliorate the behavioral deficit induced by stress (Figure 9).

## Discussion

Numerous studies have found that stress is a crucial regulatory factor for cognitive function, which can lead to poor outcomes including depression and cognitive impairment (Sandi and Pinelo-Nava, 2007; Lupien et al., 2009; Aznar and Knudsen, 2011). As a major structure of the brain associated with cognition and mood, the hippocampus is extremely susceptible to chronic stress exposure (Lupien et al., 2009). Herein, our results showed that repeated stress exposure for seven consecutive days severely impaired the learning and memory function of SD rats. Meanwhile, repeated stress exposure was found to significantly impair dendritic spine plasticity, as well as increase the NMDA receptor level in the hippocampal CA3 region. Conversely, cognitive dysfunction and impairment of the dendritic spine plasticity induced by repeated stress exposure were partially reversed after administrating an inhibitor of the NMDA receptor GluN2B.

Compared with chronic unpredictable stress, chronic restraint stress exposure more significantly induced neuronal dendritic atrophy in the hippocampal CA3 region (Vyas et al., 2002), as shown by significantly decreased dendrite length and branching number in the hippocampal CA3 region. NMDA receptors play critical roles in neuronal plasticity and memory abilities (Scannevin and Huganir, 2000; Newcomer and Krystal, 2001). Some studies have indicated that chronic restraint stress resulted in an increase in glutamate release (Gilad et al., 1990; Lowy et al., 1993), which may trigger the morphologic changes of neuronal synapse related to stress via activation of an NMDA receptor (Magariños and McEwen, 1995). NMDA receptor-mediated excitotoxicity induced by the excessive release of glutamate (Tapia, 1996) is considered to be one of the major underlying mechanism for atrophy of neuronal dendrites, which is caused by chronic exposure to restraint stress. Furthermore, Christian et al. (2011) found that selective deletion of the NMDA receptor in CA3 pyramidal neurons restricted dendrites retraction, which indicated that chronic stress-induced retraction of the hippocampal neuron dendrites requires NMDA receptors of the hippocampal CA3 region. Consistent with previous reports, we also found that chronic restraint stress exposure significantly reduced the density of dendritic spines and increased the NMDA receptor protein level in the hippocampal CA3 region, which is probably associated with NMDA receptor-mediated neuroexcitotoxicity.

The increased protein level of the NMDA receptor may induce excitatory neurotoxicity, which plays a vital role in neuronal synaptic degeneration (Donohue et al., 2006; Huang et al., 2015; Bhagya et al., 2017). LTP and LTD are the two most widely used neurophysiological measures, which are considered to be the foundation of learning and memory (Malenka and Bear, 2004). One study found that either glucocorticoid-induced or stress exposure for 14 consecutive days showed impairment of LTP (Park et al., 2015). Moreover, other researchers found that behavioral stress through a blockade of glutamate uptake, led to the activation of an NMDA receptor, which impaired LTP and facilitated the LTD of a hippocampal CA1 neuron (Yang et al., 2005). In addition, chronic immobilization stress was found to stably enhance the metabotropic glutamate receptor-dependent LTD in the hippocampal CA1 region (Sengupta et al., 2016). In our study, chronic repeated restraint stress impaired the neuronal LTP and facilitated the induction of neuronal LTD in the hippocampal CA3 region. Meanwhile, we also noticed that AP-5 (an NMDA receptor inhibitor) pre-intervention failed to significantly ameliorate the impairment of the hippocampal CA3 LTP, indicating that impairment of LTP caused by repeated restraint stress exposure probably involves other signaling pathways except NMDA receptors, which needs further investigation. However, the enhanced effect of LTD induced by repeated restraint stress exposure was blocked after AP-5 pre-intervention, suggesting that repeated restraint stress-induced LTD was partly mediated by the NMDA receptor.

To further study the underlying mechanism of enhancement of LTD induced by repeated restraint stress exposure, we used PEAQX (GluN2A inhibitor) and Ro25-6981 (GluN2B inhibitor) as a pre-intervention. We found that the GluN2A-inhibition by PEAQX pre-intervention failed to affect the induction and maintenance of LTD in rats exposed to repeated stress, while the induction of LTD was blocked by Ro25-6981 (GluN2B inhibitor). Massey et al. reported that the activation of GluN2A-containing NMDA receptors was required to induce cortical LTP, while the activation of GluN2B-containing NMDA receptors was required to induce cortical LTD (Massey et al., 2004). Thus, our present findings suggest that repeated restraint stress-induced LTD was probably mediated through GluN2B activation.

Cumulative works indicate that chronic stress markedly affects the hippocampal morphology. Repeated stress causes the shortening and debranching of dendrites in the hippocampal CA3 region and suppresses neurogenesis of DG granule neurons, reduces hippocampal DG-LTP, and induces atrophy of hippocampal DG (McEwen, 2000a; Nasca et al., 2015; Zhou et al., 2018). In the CA1 area, the structural changes reported after chronic stress include alterations in the lengths of the terminal dendritic segments of pyramidal cells, reduction of dendritic spine density, and hippocampal CA1-LTP (Huang et al., 2015; Bhagya et al., 2017). The induced structural and functional changes in different hippocampal regions depend upon the type, intensity, and duration of the stressor. Also, the age of the suffered animal, for example childhood, adolescence, adulthood or aging has an impact on brain structures involved in cognition. Animal genus (rat or mouse) or strain (SD or Wistar rat) is another important factor.

By the way, chronic stress-induced hippocampal neuroanatomical and functional changes through glutamate-mediated excitotoxicity, which imply that increased levels of surface NMDA receptors, not total levels, may correlate with neurotoxicity in chronic restraint stress models. In a future study, we will investigate the NMDA receptor level on surface membranes and the underlying mechanisms.

In conclusion, chronic repeated restraint stress exposure-induced cognitive dysfunction is mediated through impaired dendritic plasticity, as well as an increased level of NMDA receptors in the CA3 area of the hippocampus in SD rats.

## Data Availability Statement

The raw data supporting the conclusions of this article will be made available by the authors, without undue reservation.

## Ethics Statement

All animal experimental procedures were conducted according to the “Policies on the Use of Animals and Humans in Neuroscience Research,” and approved by the Ethics Committee of Tongji Medical College, Huazhong University of Science and Technology.

## Author Contributions

GL, D-JL, and R-HM designed the research. DS, GZ, H-XC, YH, X-YH, TL, XL, and QL performed the research. QW and DK analyzed the data. GL and R-HM wrote the manuscript. All authors contributed to the article and approved the submitted version.

## Conflict of Interest

The authors declare that the research was conducted in the absence of any commercial or financial relationships that could be construed as a potential conflict of interest.
